# *Eimeria bovis*-triggered neutrophil extracellular trap formation is CD11b-, ERK 1/2-, p38 MAP kinase- and SOCE-dependent

**DOI:** 10.1186/s13567-015-0155-6

**Published:** 2015-03-05

**Authors:** Tamara Muñoz-Caro, Sandra Jaqueline Mena Huertas, Ivan Conejeros, Pablo Alarcón, María A Hidalgo, Rafael A Burgos, Carlos Hermosilla, Anja Taubert

**Affiliations:** Institute of Parasitology, Justus Liebig University Giessen, Schubertstraße 81, 35392 Giessen, Germany; Laboratory of Inflammation Pharmacology, Institute of Pharmacology and Morphophysiology, Faculty of Veterinary Science, Austral University of Chile, Valdivia, 5110566 Chile; Department of Biology, University of Nariño, Pasto, 520002 Colombia

## Abstract

*Eimeria bovis* is an important coccidian parasite that causes high economic losses in the cattle industry. We recently showed that polymorphonuclear neutrophils (PMN) react upon *E. bovis* sporozoite exposure by neutrophil extracellular trap (NET) formation. We focused here on the molecular mechanisms that are involved in this process. The sporozoite encounter led to an enhanced surface expression of neutrophil CD11b suggesting a potential role of this receptor in *E. bovis*-mediated NETosis. Antibody-mediated blockage of CD11b confirmed this assumption and led to a significantly decreased sporozoite-triggered NET. In addition, *E. bovis*-induced NETosis was found to be Ca^2+^-dependent since the inhibition of store-operated calcium entry (SOCE) significantly diminished NET. Furthermore, NADPH oxidase, neutrophil elastase (NE) and myeloperoxidase (MPO) were confirmed as key molecules in sporozoite-triggered NETosis, as inhibition thereof blocked parasite-triggered NET. PMN degranulation analyses revealed a significant release of matrix metalloprotease-9 containing granules upon sporozoite exposure. We further show a significantly enhanced phosphorylation of ERK1/2 and p38 MAPK in sporozoite-exposed PMN indicating a key role of this signaling pathway in *E. bovis*-mediated NETosis. Accordingly, ERK 1/2 and p38 MAPK inhibition led to a significant decrease in NET formation. Finally, we demonstrate that sporozoite-induced NETosis is neither a stage-, species-, nor host-specific process.

## Introduction

*Eimeria bovis* is an important intracellular apicomplexan parasite of cattle, causing severe haemorrhagic diarrhoea (typhlocolitis) especially in calves leading to high economic losses worldwide [1]. Polymorphonuclear neutrophils (PMN) appear to play a pivotal role in *E. bovis* defense. This leukocyte population was identified in parasitized intestine of *E. bovis*-infected calves [2] and PMN have been shown to interact directly with *E. bovis* stages and antigen, resulting in direct elimination or production of pro-inflammatory cytokines, chemokines and iNOS upon encounter [3]. Additionally, PMN were shown to adhere to *E. bovis-*infected host endothelial cells [4] and their phagocytic and oxidative burst activities were enhanced in response to *E. bovis* sporozoites in vitro or ex vivo during infection [3]. Furthermore, we recently identified sporozoites of *E. bovis* as potent inducers of neutrophil extracellular traps (NET) [5]. Overall, NET formation has been described as a novel form of cell death called ETosis which is distinct from apoptosis, autophagy and necrosis leading to extracellular entrapment and eventually the killing of pathogens [6,7]. NET have been shown to be involved in several infections caused by bacteria, viruses and fungi [8-11]. However, little attention has been paid to the role of NET in the early host innate immune response against protozoan parasites [12,13]. By now, NET formation has been described to be induced by some protozoan parasites, such as *Leishmania* spp. [14], *E. bovis, Toxoplasma gondii, Plasmodium falciparum, E. arloingi*, and *Besnoitia besnoiti* [5,12,15-17].

NETosis is an NADPH oxidase-mediated ROS-dependent mechanism [7-11] which causes the expulsion of a mixture of nuclear and cytoplasmic granule contents leading to the formation of fiber-like structures being decorated with histones and granular molecules, such as neutrophil elastase (NE) and myeloperoxidase (MPO) amongst others [7,10,11]. On the mechanistic level, parasites are immobilized within these DNA-rich structures and may be killed via high local concentrations of antimicrobial molecules [12,13]. Consequently, some cases of parasite-triggered NETosis have been described as ROS-, NE- or MPO-dependent [5,12,16,17].

So far, few pathogen-derived molecules triggering NETosis have been identified such as bacterial toxins [18], virus [19] or surface lipophosphoglycans of *Leishmania* [14]. In addition, little data are reported on NET-associated PMN-derived ligands and Ca^2+^-mediated signalling pathways [20]. As such, CD11b and CD18 are described as being involved in *Candida albicans* [21] or *Mannheimia haemolytica*-mediated NETosis [18]. Recently Ca^2+^-dependent ETosis has been reported [20]. In the case of parasite-triggered NETosis, no data on PMN ligands or Ca^2+^-dependency are available, so far. This work focuses on molecular mechanisms involved in *E. bovis*-induced NET formation. We here demonstrate that respective NETosis depends on granulocyte NE and MPO activities and on the activation of an ERK1/2- and p38-related signaling pathway. Furthermore store-operated Ca^2+^ entry and CD11b receptor binding is involved in *E. bovis*-triggered NETosis.

## Materials and methods

### Parasite

The *E. bovis* (strain H) used in the present study was maintained by passages in Holstein–Friesian calves for oocyst production as described by Hermosilla et al. [22]. Calves were infected orally with 5 × 10^4^ sporulated *E. bovis* oocysts. Collection of oocysts, oocyst sporulation and excystation of sporozoites were performed as previously described [22]. Free-released sporozoites were washed three times with sterile PBS (400 × *g*, 10 min), counted in a Neubauer haemocytometer chamber and thereafter suspended at final concentrations of 2.5 × 10^5^ − 10^6^ sporozoites/mL in culture medium (RPMI 1640, 1% penicillin/streptomycin, v/v; Gibco) free of fetal calf serum (FCS) until further experimental use. In addition, sporulated *E. arloingi* oocysts (strain A) [16], *Isospora suis* oocysts (kindly provided by Anja Joachim, University of Veterinary Medicine of Vienna, Austria) and *Toxoplasma gondii* oocysts (kindly provided by Anja Joachim, University of Veterinary Medicine of Vienna, Austria and Peter Deplazes, Faculty of Veterinary Medicine, University of Zurich, Switzerland) were used for comparative analyses on parasite species-specific NET induction. Sporozoite excystation of the latter parasite species was performed according to Pinckney et al. [23] as well as Freyre and Falcon [24].

### Isolation of bovine PMN

Adult dairy cows (*n =* 3) were bled by puncture of the jugular vein and blood was collected in 50 mL plastic tubes (Nunc) containing 0.1 mL heparin (Sigma-Aldrich) as anticoagulant. Heparinized blood was diluted in an equal amount of PBS containing 0.02% EDTA (Sigma-Aldrich), layered on Biocoll Separating Solution® (Biochrom AG) and centrifuged at 800 × *g* for 45 min. After removal of the plasma and PBMC layer, the pellet was suspended in 25 mL distilled water and gently shaken for 40 s to lyse erythrocytes. Osmolarity was immediately re-adjusted by adding 3 mL of Hanks Salt Solution (HBSS 10 x, Biochrom AG). PMN were washed twice, re-suspended in RPMI 1640 medium (Gibco), counted in a Neubauer haemocytometer chamber and subsequently incubated at 37 °C and 5% CO_2_ atmosphere for at least 30 min before use.

### Flow cytometric analysis of *Eimeria bovis* sporozoites-induced CD11b surface expression on bovine PMN

PMN were incubated with CFSE-stained *E. bovis* sporozoites (1:1 ratio, 30 min, 37 °C) in HBSS containing 0.9 mM CaCl_2_. For positive controls, PMN were treated with platelet activating factor (PAF 100 nM, 15 min, 37 °C; Calbiochem). For negative controls, non-treated and non-exposed PMN were used. After incubation, cells were pelleted (300 × *g*, 20 °C, 6 min), resuspended in 200 μL HBSS and incubated with anti-CD11b antibodies coupled to allophycocyanin (APC; clone M1/70; 20 min, in the dark; BD Pharmingen) and washed at 300 × *g*, 20 °C, 6 min. Thereafter, the cells were analyzed using a FACS Canto II cytometer (BD Biosciences, San Diego, CA, USA). The data were displayed as plots of forward versus side light scatter. The mean fluorescence of APC was determined from a minimum of 10^4^ cells using BD FACS Diva 6.1 software (BD Biosciences, San Diego, CA, USA).

### Inhibition of store-operated calcium entry (SOCE)

Bovine PMN were pre-incubated with the SOCE inhibitor 2-aminoethoxydiphenyl borate (2-APB) for 15 min at concentrations of 50 and 100 μM in HBSS medium prior to exposure to *E. bovis* sporozoites in a 1:2 ratio (2 × 10^5^ PMN: 4 × 10^5^ sporozoites, 60 min, 37 °C).

For NET quantification, 50 μL of micrococcal nuclease buffer containing 0.1 U/μL micrococcal nuclease (both New England Biolabs) were added to each well and incubated (15 min, 37 °C). Afterwards the samples were centrifuged (300 × *g*, 5 min) and the supernatants were transferred into a 96-well flat-bottom plate (100 μL per well in duplicates). Fifty microliters of Pico Green® (1:200 dilution in 10 mM Tris base buffered with 1 mM EDTA) were added to each sample and the samples were incubated (4 min, in the dark). NET formation was determined by spectrofluorometric analysis at an excitation wavelength of 484 nm and an emission wavelength of 520 nm using an automated plate monochrome reader (Varioskan Flash®; Thermo Scientific). For negative controls, PMN in plain medium were used. For positive controls, stimulation with zymosan (1 mg/mL) was used.

### Immunoblotting for the detection of phosphorylated ERK1/2 and p38 MAPK

PMN (5 × 10^6^ in HBSS containing 0.9 mM CaCl_2_) were exposed to *E. bovis*-sporozoites (5 × 10^6^) for 15 and 30 min at 37 °C in a final volume of 500 μL. For positive controls, PMN were stimulated with PAF (100 nM). Thereafter, total protein was extracted as described previously by Hidalgo et al. [25] and 40 μg total protein were analyzed by a 10% SDS-PAGE. Immunoblotting was performed according to a protocol previously described by Hidalgo et al. [26]. Antibodies directed against phospho-p38 MAPK and phospho-ERK1/2 (Cell Signalling, Beverly, MA, USA) were used according to the instructions provided by the manufacturer. Anti-mouse HRP-conjugated antisera (Santa Cruz Biotechnology, USA) were used as secondary antibodies (2 h of incubation, RT, in constant agitation). Signals were detected using an enhanced chemiluminescence system (Western Lightning® Plus-ECL; Perkin-Elmer, USA). After signal detection and documentation, the bound antibodies were removed by stripping the membranes (100 mM 2-mercaptoethanol; 2% SDS; 62.5 mM Tris-HCl, pH 6.7, for 2 h at 50 °C with agitation, followed by several washes with TBS-Tween 0.1%) according to Hidalgo et al. [25] and each membrane was re-probed with an antibody recognizing total p38 MAPK (p38 MAPK anti rabbit antibody; Cell signaling technology) and ERK1/2 (rabbit polyclonal IgG; Santa Cruz Biotechnology, USA). The samples were then further processed as described above. The intensities of each band were analyzed using the Software Image J and the signals were normalized to total ERK1/2 for p-ERK1/2 and to total p38 for p-p38.

### NET inhibition assays using ERK1/2, p38 MAPK inhibitors and functional CD11b monoclonal antibodies

For the inhibition of ERK 1/2 and p38 MAPK the following inhibitors were used respectively: UO126 (50 μM; Sigma-Aldrich) and SB 203580 (10 μM; Sigma-Aldrich). Therefore, PMN were preincubated with the inhibitors for 30 min at RT in HBSS-buffer without phenol red (Gibco). CD11b was blocked via pre-incubation in mouse anti bovine CD11b monoclonal antibodies (MCA1425, diluted 1:5 in PBS; AbD Serotec). For antibody control, we used an irrelevant antibody at an identical concentration (anti-bovine CD4, AbD Serotec). Then, PMN were exposed to viable *E. bovis-*sporozoites (1:2 ratio: 2 × 10^5^ PMN + 4 × 10^5^ sporozoites) for 60 min at 37 °C. Thereafter extracellular DNA was quantified as described above. All measurements were achieved using an automated monochrome reader (Varioskan Flash; Thermo Scientific). For positive controls, zymosan (Sigma-Aldrich) was used in a final concentration of 0.5 mg/mL.

### Visualization of NET and detection of histones (H3), neutrophil elastase (NE) and myeloperoxidase (MPO) in NET structures

Bovine PMN were incubated with *E. bovis* sporozoites (ratio 1:1; 30 min) on poly-_L_-lysine-treated coverslips and fixed [4% (w/v) paraformaldehyde, Merck, 20 min in the dark]. NET structures were visualized by staining extracellular DNA with 5 mM Sytox Orange dye (Invitrogen) for 10 min at RT according to Martinelli et al. [27]. For the visualization of sporozoites within NET structures, sporozoites were stained with 5(6)-carboxyfluorescein diacetate succinimidyl ester (CFSE, 7.5 μM, 37 °C, 30 min; Invitrogen) according to Hermosilla et al. [28] prior to PMN exposure. After fixation of the cells and three washings in sterile PBS, the samples were mounted in anti-fading buffer (Mowiol®, Sigma-Aldrich) and stored (4 °C, in the dark) until further use. For the detection of histones, MPO and NE within NET structures the following specific antibodies were used: anti-histone monoclonal antibodies [rabbit (E173) anti-bovine histone H3, phospho S10 DyLight® 488, 1:100; ab139848, Abcam], anti-MPO antibodies (rabbit anti-bovine MPO, Alexa Fluor 488, 1:200; ABIN906866) and anti-NE antibodies (rabbit anti-human NE, 1:200; AB68672, Abcam). Therefore, the samples were washed three times with PBS, blocked with BSA [1% (w/v) in PBS, 30 min, RT, Sigma-Aldrich] and reacted with anti-histone, anti-NE or anti-MPO antibodies [1 h, RT, in the dark for bovine anti-histone (H3); 24 h, RT, in the dark for both anti-MPO and anti-NE antibodies]. The samples were then gently washed in PBS and mounted in anti-fading buffer (Mowiol®, Sigma-Aldrich). Visualization was achieved using an inverted Olympus® IX81 fluorescence microscope equipped with a digital camera.

### Measurements of NADPH oxidase, NE and MPO enzymatic activities and inhibition of these enzyme activities

For NADPH oxidase-, NE- and MPO-inhibition assays, the following inhibitors were used: the NADPH oxidase inhibitor diphenylene iodonium (DPI, 10 μM, Sigma-Aldrich); the NE inhibitor Suc-Ala-Ala-Pro-Val chloromethyl ketone (CMK; 1 mM, Sigma-Aldrich), according to Scapinello et al. [29] and the MPO inhibitor 4-aminobenzoic acid hydrazide (ABAH; 100 μM, Calbiochem), according to Parker et al. [30]. In brief, PMN and sporozoites (1:1 ratio, *n* = 3) were incubated (30 min, 37 °C) in HBSS-buffer without phenol red (Gibco) for positive controls. In parallel, PMN were pre-incubated with the corresponding inhibitors for 30 min at RT prior to exposure to viable *E. bovis-*sporozoites (1:1 ratio, 30 min, 37 °C). To estimate maximum values of extracellular DNA, PMN were lysed by Triton-X 100 treatment (0.1%; Sigma-Aldrich). To block NET formation, 90 U of DNase I (Roche Diagnostics) were supplemented 15 min before the end of incubation period. NET were quantified via Pico Green®-DNA staining as described above.

NADPH oxidase, NE and MPO enzymatic activities were measured using respective substrates: 10 μg/mL DCFH-DA (Sigma-Aldrich); 3 mg/mL of the NE chromogenic substrate MeoSuc-Ala-Ala-Pro-Val-chloromethyl-ketone (Sigma-Aldrich) and 50 μM Amplex red (Invitrogen), respectively. ROS production was measured by oxidation of DCFH-DA to fluorescent DCF according to Conejeros et al. [31]. The relative fluorescence units (RFU) were recorded at 15 min intervals for a period of 30 min applying 485 nm excitation and 530 nm emission wavelengths. NE activity was measured every 10 min for 30 min at 410 nm wavelength and MPO-peroxidase activity was measured every 10 min for 30 min in 571-585 nm fluorescence ranges. All measurements were achieved using an automated monochrome reader (Varioskan Flash; Thermo Scientific). As positive control, zymosan (Sigma-Aldrich) was used at a final concentration of 0.5 mg/mL.

### Determination of matrix metalloproteinase 9 (MMP-9) activities in PMN supernatants

PMN (10^6^/500 μL HBSS/0.9 mM CaCl_2_) were exposed to equal numbers of *E. bovis* sporozoites (15 and 30 min, 37 °C). Stimulation of PMN with PAF (100 nM, 5 min, 37 °C) was used for positive controls. PMN cultivated in plain medium were used for negative controls. After incubation, the cells were centrifuged (600 × *g*, 6 min) and the supernatants were assayed for gelatinase activity by zymography. Therefore, substrate gel electrophoresis was performed using the method described by Conejeros et al. [32]. Briefly, 10 μL of supernatant/slot were loaded on polyacrylamide gels (10%, 0.75 mm thickness) containing 0.28% of gelatin (Sigma-Aldrich). In parallel a recombinant MMP-9 standard (Sigma-Aldrich) and a molecular mass marker (Fermentas International Inc., Canada) were loaded as reference samples. The gels were run at 200 V for 1 h in a Bio-Rad Mini Protean II chamber (Bio-Rad Laboratories, CA, USA). Thereafter, the gels were incubated twice in Triton X-100 (2.5%, under constant shaking, RT, 30 min) and overnight at 37 °C in reaction buffer (100 mM Tris, pH 7.5; 10 mM CaCl_2_). The gels were stained in Coomassie Brilliant Blue R-250 (Winkler, Santiago, Chile; 0.5% in acetic acid : methanol : water = 1 : 3 : 6). MMP-9 (gelatinase B) enzymatic activity present in the test samples were determined according to the degree of gelatin degradation (visible as clear bands of 82 kDa) relative to the MMP-9-control by means of band intensity measurements (applying ImageJ 1.35 s software).

### Host cell infection and production of viable *Eimeria bovis* merozoites I

Primary bovine umbilical vein endothelial cells (BUVEC) were isolated from umbilical cord veins, according to the method of Hermosilla et al. [4]. Confluent BUVEC monolayers (*n* = 3) were infected with 2.5 × 10^5^ freshly excysted *E. bovis*-sporozoites suspended in modified ECGM [ECGM (PromoCell) supplemented with 70% (v/v) M199 (Gibco), 2% FCS (Gibco) and 1% penicillin (Sigma-Aldrich)] per 25 cm^2^ flask (Greiner). The cells were fed 24 h after infection and thereafter every third day with modified ECGM. To control for *E. bovis* macromeront development and the release of merozoites I, infected BUVEC cultures were analyzed daily over a period of 22 days using a phase-contrast inverted microscope (IX81 microscope®, Olympus). Free-released merozoites I were harvested from the supernatant of infected BUVEC monolayers (400 × *g*, 5 min) and washed twice in PBS. The merozoites I were counted in a Neubauer chamber haemocytometer.

### Scanning electron microscopy (SEM)

Bovine PMN were incubated with *E. bovis* sporozoites*,* merozoites I or oocysts at a ratio of 1:1 for 90 min on poly-_L_-lysine (Sigma-Aldrich) pre-coated coverslips. After incubation, the samples were fixed (2.5% glutaraldehyde in 0.1 M cacodylate buffer, 15 min, all Merck) and washed with 0.1 M cacodylate buffer. The samples were post-fixed in 1% osmium tetroxide (Merck) in 0.1 M cacodylate buffer, washed three times in distilled water, dehydrated in ascending ethanol concentrations, critical point dried by CO_2_-treatment and sputtered with gold. Specimens were examined using a Philips XL30 scanning electron microscope at the Institute of Anatomy and Cell Biology, Justus Liebig University Giessen, Germany.

### Quantification of NET derived from PMN and parasites from different origins

NET formation was quantified using Pico Green® (Invitrogen), a DNA-binding dye. In order to quantify *E. bovis* sporozoite- or merozoite I-induced NET formation, bovine PMN (*n* = 3-5) were re-suspended in serum-free medium RPMI 1640 and incubated (all at 37 °C) with *E. bovis-*sporozoites in a 1:2 ratio (2 × 10^5^ PMN: 4 × 10^5^ sporozoites, 60 min), with *E. bovis-*merozoites I in a 1:1 ratio (1 × 10^5^ PMN: 1 × 10^5^ merozoites, 90 min), with oocysts (5 × 10^4^, 60 min, 1:1) and with sporozoites of *E. arloingi* (*n* = 3; 1 × 10^5^ PMN: 1 × 10^5^ sporozoites, 60 min), *Isospora suis* (*n* = 3; 1 × 10^5^ PMN: 1 × 10^5^ sporozoites; 60 min) and *T. gondii* (*n* = 5; 1 × 10^5^ PMN: 1 × 10^5^ sporozoites; 60 min). In addition, PMN isolated from different host species origin (caprine, horse and dog) were incubated with *E. bovis* sporozoites in a 1:2 ratio (2 × 10^5^ PMN: 4 × 10^5^ sporozoites, 60 min at 37 °C). For positive controls, zymosan was used at a final concentration of 0.5 mg/mL. Plain RPMI 1640 medium served as negative controls. NET-formation was determined based on Pico Green®-DNA staining as described above.

### *Eimeria bovis* sporozoite entrapment assay

For the quantification of sporozoite entrapment within NET structures we followed the method described by Köckritz-Blickwede et al. [8] with slight modifications. Briefly, PMN (2.5 × 10^5^/96-well) were pre-activated by zymosan treatment (30 min, 37 °C). Meanwhile, *E. bovis-*sporozoites were stained with FITC dye (0.2 mg/mL, 30 min, on ice, in the dark, Invitrogen) and washed twice in PBS (3000 × *g*, 10 min). Thereafter, zymosan-stimulated PMN were exposed to FITC-labeled *E. bovis* sporozoites (512 × *g*, 10 min; subsequent incubation for 30 min at 37 °C) in ascendant ratios (0.5:1;1:1 and 1:2). Non-exposed sporozoites were used for controls (1:1; 1:2). The samples were washed twice in RPMI 1640 medium and measured for fluorescence intensity at 485/538 nm wavelength. The percentage of entrapment was calculated as follows: [(A485/538 nm sporozoites exposed to PMN)/(A485/538 nm non-exposed sporozoites)] × 100%.

### Ethics statement

All animal procedures were performed according to the Justus Liebig University Animal Care Committee guidelines, approved by the Ethic Commission for Experimental Animal Studies of the State of Hesse (Regierungspräsidium Giessen) and in accordance to the current German Animal Protection Laws. Identification number of animal care and project licence: GI 18/10-Nr.A51/2012 544_AZ952 (*Eimeria bovis*-oocyst production); GI 18/10-Nr.A9/2012 521_AZ877 (Bovine blood samples). European Animal Welfare Legislation: ART13TFEU.

### Statistical analysis

Co-culture/stimulation conditions were compared by one- or two-factorial analyses of variance (ANOVA) with repeated measurements in order to compare co-culture/stimulation conditions. All analyses were performed with the GraphPad Prism® 6 software. Differences were regarded as significant at a level of *p* ≤ 0.05 (*); *p* ≤ 0.01 (**); *p* ≤ 0.001 (***).

## Results

### Exposure to *E. bovis* sporozoites up-regulates neutrophil CD11b surface expression on bovine PMN and triggers NET formation in a CD11b-dependent manner

**Figure 1:**
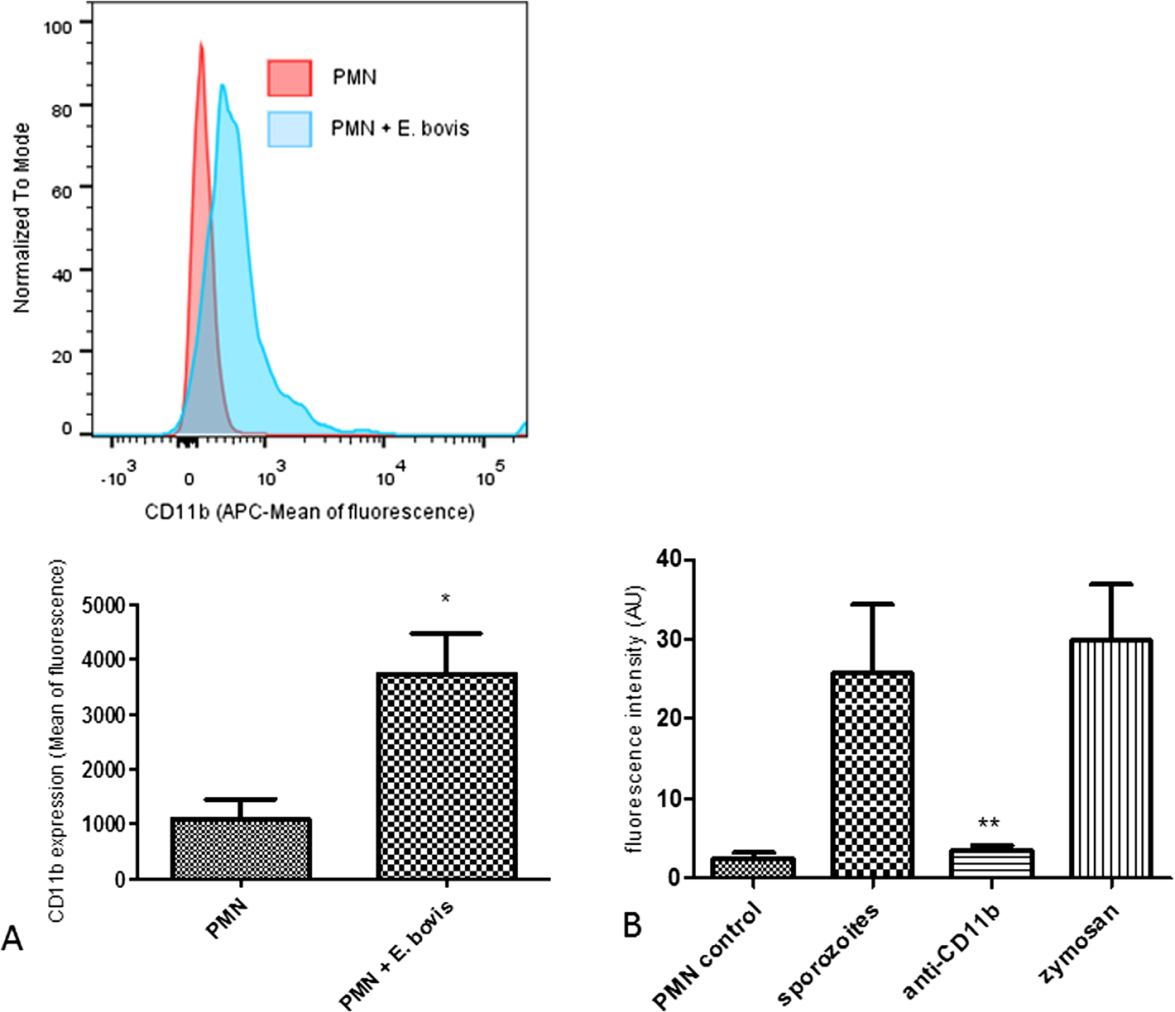
**CD11b surface expression and CD11b-dependent-NET formation of bovine** ***E. bovis*** **sporozoite-exposed PMN. (A)** Bovine PMN were exposed to CFSE-labeled sporozoites (1:1) and subjected to FACS analysis probing with an antibody directed against CD11b. Non-exposed PMN in plain medium served as negative controls. The histograms represent the shift in the mean fluorescence of the PMN population analyzed and are representative of the experiments performed. **(B)** NET formation in the presence of anti-CD11b antibodies. Bovine PMN were pre-treated with anti-CD11b prior to sporozoite exposure. Stimulation with zymosan served as a positive control; PMN in plain medium were used for negative controls.

Since no data are available on PMN-derived receptors being involved in *E. bovis*-mediated NETosis, we here analyzed whether sporozoite exposure would affect CD11b surface expression of bovine PMN. Applying FACS methodology, a significant parasite-triggered increase of CD11b surface expression was shown when compared to non-exposed PMN (*p* ≤ 0.05, Figure 1A). In addition, blockage of CD11b via specific antibodies led to a significant reduction in sporozoite-mediated NET formation when compared to antibody-free controls (*p* ≤ 0.01; Figure 1B). We also did not observe any significant NET induction using our irrelevant antibody control. Thus, these results suggest CD11b as a potential candidate for neutrophil receptors being involved in NET formation.

### *Eimeria bovis*-induced NET formation is store-operated calcium entry (SOCE)-dependent

**Figure 2:**
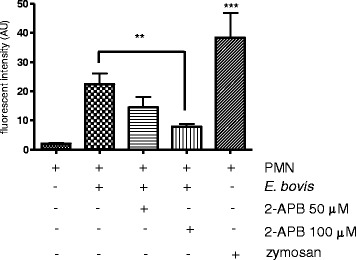
**Influence of store-operated calcium entry (SOCE) on** ***E. bovis*** **sporozoites-induced NET formation.** PMN were treated with the SOCE-inhibitor 2-APB prior to exposure to *E. bovis* sporozoites. For negative controls, non-treated PMN were used. For positive controls, zymosan stimulation was used. NET formation was quantified based on DNA-derived fluorescence intensities using an automate plate reader.

As previously shown in the bovine system, ROS production is a Ca^2+^-dependent process [32]. Since sporozoite-triggered NETosis was recently proven to be ROS-dependent [5], we here analyzed whether the presence of the Ca^2+^ (SOCE) inhibitor 2-APB [31,32] would influence sporozoite-mediated NETosis. The fact that treatment with 100 μM 2-APB resulted in a significant reduction of *E. bovis* sporozoite-induced NET when compared with non-treated but sporozoite-exposed PMN (*p* ≤ 0.01, Figure 2), suggests a Ca^2+^/SOCE-dependent process. Overall, stimulation of PMN with zymosan revealed to be a potent SOCE inducer compared to the negative control (*p* ≤ 0.001; Figure 2).

### Sporozoite-induced NETosis is a ROS-, NE- MPO-dependent process

**Figure 3:**
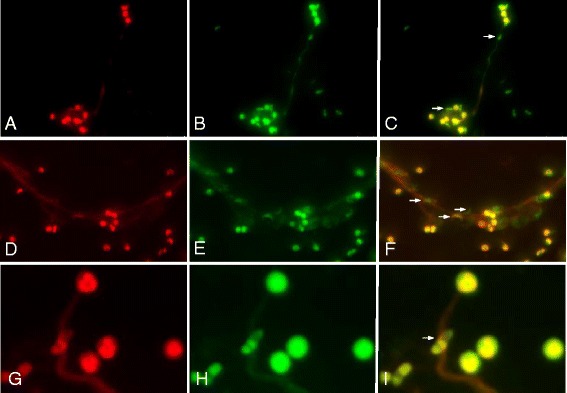
**Co-localization of DNA with histones (H3), neutrophil elastase (NE) and myeloperxodase (MPO) in** ***E. bovis*** **sporozoite-induced NET structures.** Co-cultures of bovine PMN and *E. bovis* sporozoites and oocysts were fixed, permeabilized and stained for DNA using Sytox Orange (red: **A**, **D**, **G**) and probed for histone 3 (H3) (green: **B**), MPO (green: **E**) and NE (green: **H**) using anti-H3, anti-NE and anti-MPO antibodies jointly with adequate conjugate systems. Areas of respective co-localization (merges) are illustrated in **C**, **F**, **I**. Photomicrographs are of representative cells from 3 independent experiments.

**Figure 4:**
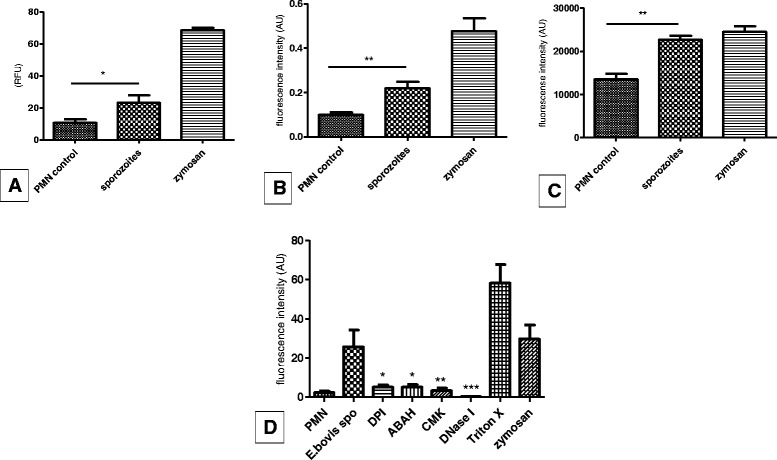
***E. bovis*** **sporozoite-induced NETosis in bovine PMN is a NADPH oxidase-, NE- MPO-dependent process. (A-C)** NADPH oxidase, NE and MPO enzymatic activities in *E. bovis* sporozoite-exposed PMN. PMN were exposed to *E. bovis* sporozoites and enzymatic activities of NADPH oxidase **(A)**, NE **(B)** and MPO **(C)** were measured using via DCFH-DA oxidation, NE-chromogenic substrate analysis and using Amplex red reagent, respectively. **(D)** Sporozoite-triggered NET formation after inhibition of NADPH oxidase (via DPI), NE (via CMK) and MPO (via ABAH). For inhibition and maximum DNA release controls, DNase I and Triton X treatments were used, respectively. Stimulation with zymosan served as a positive control (1 mg/mL) and PMN in plain medium as a negative control.

Experiments applying specific antibodies against H3, NE and MPO alongside with DNA-staining proved NET structures (estimated via extracellular DNA-positive fibres) as co-localizing with histone H3-, NE- and MPO-positive signals indicated a pivotal role of these molecules in sporozoite-triggered NETosis and confirmed classical NET characteristics (Figure 3). In addition, PMN exposure to *E. bovis* sporozoites resulted in a significant up-regulation of NADPH oxidase-, NE- and MPO- enzymatic activities (*p* ≤ 0.05 for NADPH oxidase; *p* ≤ 0.01 for NE and MPO; Figures 4A-C). Furthermore, we confirmed the key role of these enzymes since inhibition of NADPH oxidase, NE and MPO led to significantly reduced sporozoite-triggered NETosis when compared to non-treated controls (Figure 4D; DPI and ABAH: *p* ≤ 0.05; CMK: *p* ≤ 0.01). As expected, DNase I treatments led to a significant NET resolution (*p* ≤ 0.001, Figure 4D). Total DNA release from PMN was controlled by Triton X treatments revealing a proportion of up to 43% of PMN to be involved in sporozoite-mediated NETosis. Overall, these data confirm the relevance of NADPH oxidase, NE and MPO in *E. bovis* sporozoite-induced NET formation.

### *E. bovis* sporozoite exposure enhances matrix metalloprotease 9 (MMP-9) release of bovine PMN

**Figure 5:**
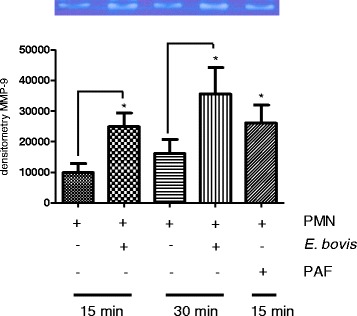
**Release of MMP-9 from PMN granule contents after sporozoite-exposure.** Bovine PMN were exposed to *E. bovis* sporozoites and the supernatants were analyzed for MMP-9 activities via zymography in gelatin-containing polyacrylamide gels and consecutive densitometric estimations. Recombinant MMP-9 was used as an internal standard. Stimulation of PMN with PAF (100 nM) served as a positive control. Each bar represents arithmetic means ± SEM of x PMN donors.

Given that tertiary granule contents of PMN also contribute to NET formation based on their antimicrobial peptide/protease activities as described by Brinkmann et al. [6], we additionally analyzed PMN supernatants for the enzymatic activity of MMP-9. Co-cultures of PMN with *E. bovis* sporozoites led to a significant increase of MMP-9 release (*p* ≤ 0.05; Figure 5) at both time points tested (15 and 30 min) indicating that tertiary granules or MMP-9 may also be involved in sporozoite-triggered NETosis. As expected, stimulation with PAF revealed to be a reliable positive control for MMP-9 release in the bovine system (*p* ≤ 0.05).

### ERK1/2- and p38 MAPK-signaling pathways are involved in *E. bovis* sporozoite-triggered NET extrusion

**Figure 6:**
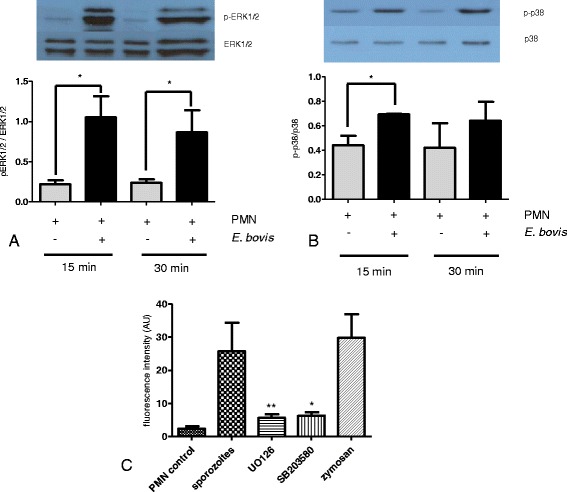
**Expression of phosphorylated ERK1/2- and p38 MAPK in sporozoite-exposed PMN and influence of their inhibition on parasite-triggered NETosis.** Total proteins of sporozoite-exposed PMN and non-exposed controls were analyzed by SDS/PAGE and immunoblot technique using specific antibodies against non-phosphorylated and phosphorylated ERK1/2 **(A)** and p38 MAPK **(B)**. Respective bands were densitometrically analyzed. Each bar represents the arithmetic means ± SEM of three PMN donors. Differences were regarded as significant at a level of *p* ≤ 0.05 compared to negative controls. **(C)** NET formation in presence of ERK 1/2 (UO126) and p38 (SB 203580) MAPK inhibitors. Stimulation with zymosan served as a positive control; PMN in plain medium as a negative control.

We analyzed whether the ERK1/2 and p38 MAPK signaling pathway is involved in sporozoite-triggered NETosis. Exposure of *E. bovis* sporozoites to PMN resulted in a fast and significant phosphorylation of ERK1/2 (*p* ≤ 0.05 after 15 and 30 min of incubation, Figure 6A) and p38 (*p* ≤ 0.05 at 15 min, Figure 6B) MAPK. Furthermore, experiments applying inhibitors of ERK1/2 and p38 MAPK confirmed the relevance of this signaling pathway since treatments with both inhibitors resulted in a significant reduction of sporozoite-mediated NET formation (UO126: *p* ≤ 0.01 and SB 203580: *p* ≤ 0.05, Figure 6C).

### Sporozoite-triggered NETosis is neither stage- nor parasite species- nor host-specific

**Figure 7:**
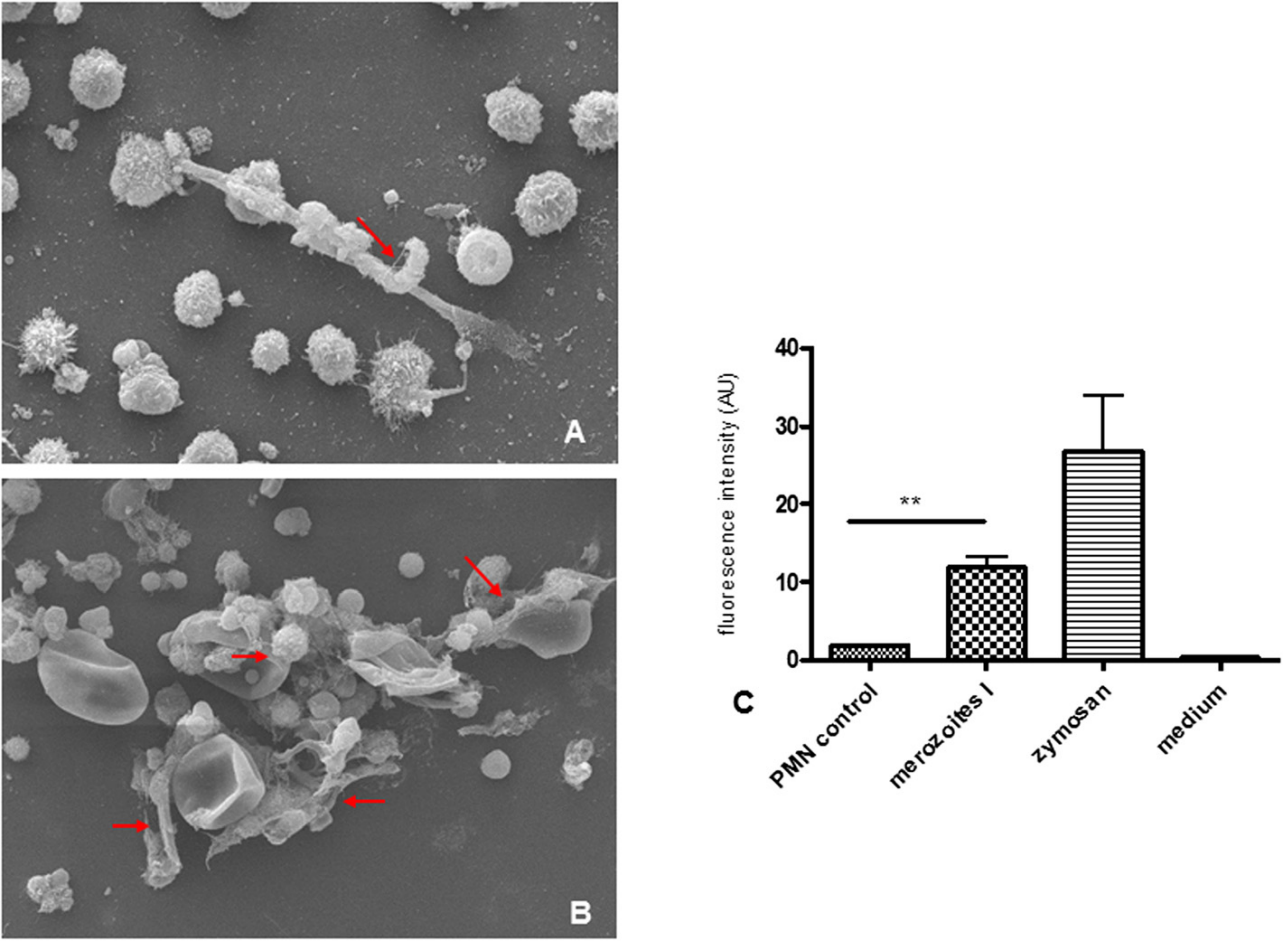
**NET formation triggered by** ***E. bovis*** **merozoites I and oocyst stages. (A)** Scanning electron microscopy (SEM) analysis of *E. bovis* merozoite I-exposed PMN. The arrow indicates a merozoite I being trapped in NET structures**. (B)** SEM analysis of *E. bovis* oocyst-exposed PMN revealing NET-like, delicate PMN-derived filaroid structures being attached to oocysts as indicated by arrows. **(C)** Quantification of *E. bovis* merozoite I-induced NET formation. PMN were exposed to *E. bovis* merozoites I, zymosan (positive control) or plain medium (negative control). NET formation was estimated by Pico Green-derived fluorescence intensities. Arithmetic means of three PMN donors, minimum and maximum.

To account for stage-specificity we tested *E. bovis* merozoites I for the capability to induce NET. As depicted in Figure 7C, merozoite I stages significantly triggered NET when compared to non-exposed controls (*p* ≤ 0.01). These data were confirmed by SEM analyses illustrating the formation of a delicate, NET-like network of thicker and thinner strands of fibres originating from dead PMN and being firmly attached to the merozoites I (Figure 7A). In addition, SEM analyses indicated NET-like structures also to be induced by oocyst stages (Figure 7B). These data clearly argue against a stage-specificity of *E. bovis*-triggered NETosis highlighting the capability of PMN to equally respond to different parasitic stages of this parasite.

**Figure 8:**
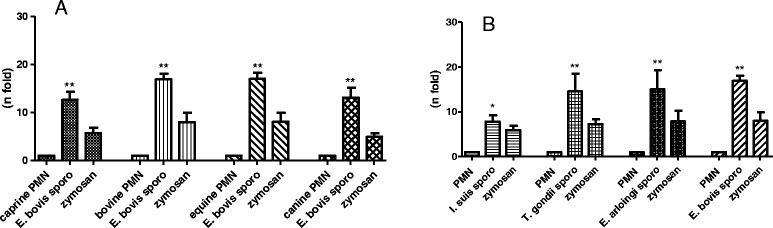
***E. bovis-*** **sporozoite triggered NETosis of PMN of different host origins and NET formation induced by different coccidian sporozoites. (A)** PMN of caprine, bovine, equine and canine origin were exposed to *E. bovis* sporozoites and respective NET formation was measured according to Pico Green-derived fluorescence intensities. **(B)** NET formation of bovine PMN in response to sporozoites of different coccidian species (*I. suis*, *T. gondii*, *E. arloingi* and *E. bovis*). In both experiments, stimulation with zymosan served as positive controls and PMN in plain medium were used for negative controls. Arithmetic means of three PMN donors, minimum and maximum.

In order to analyze whether *E. bovis*-triggered NETosis is a parasite-specific event or rather reflects a general mechanism that commonly accounts for most coccidian parasites, we analyzed NET formation of bovine PMN after exposure to the sporozoite stage of a non-bovine *Eimeria* spp. (the caprine-specific species *E. arloingi*) and of two non-*Eimeria* coccidian species (*T. gondii* and *I. suis*). The data clearly show that the sporozoites of all three parasite species equally induced significant NET formation (*E. arloingi* and *T. gondii*: *p* ≤ 0.01, *I. suis*: *p* ≤ 0.05, Figure 8B). Overall, these findings rather argue against a parasite-specific mechanism and propose NET formation as a generally valid effector mechanism against the sporozoite stage of different coccidian species.

Given that *E. bovis* is a strict host-specific parasite and, in consequence, exclusively infects bovines, we then analyzed whether *E. bovis*-mediated NET formation is a host-specific event. Therefore, PMN of different host origins (horse, goat, dog and cattle) were controlled for NET formation after exposure to *E. bovis* sporozoites. The data clearly revealed that PMN of all host types reacted by significant NET formation (*p* ≤ 0.01, Figure 8A) which argues against a host-specific reaction.

### NET structures significantly entrap *Eimeria bovis* sporozoites

In order to analyze the capacity of NET to trap viable *E. bovis* sporozoites, we established a quantitative parasite-entrapment-assay by using FITC-stained parasites and zymosan pre-activated bovine PMN as described elsewhere [17]. The experiments revealed a proportion of 43.4% of sporozoites to be immobilized in NET structures when compared to non-exposed sporozoites (data not shown). These results indicate a rather high efficacy of NET as an effector mechanism considering that almost every second parasite was entrapped and most probably hampered from host cell invasion as reported elsewhere [5].

## Discussion

NET formation depends on the assembly/activation of the NADPH oxidase complex and the resulting production of ROS [7]. Since these mechanisms were reported as Ca^2+^-mediated in the bovine system [31], it appears likely that NETosis is a Ca^2+^-dependent process, as recently demonstrated by Gupta et al. [20]. The fact that NET formation is induced by thapsigargin [33] which mobilizes Ca^2+^ from intracellular pools, also indicates a key role of Ca^2+^ in NETosis. Since SOCE was proven to play a crucial role in Ca^2+^-dependent ROS production [34] we here used 2-APB a well-known inhibitor of SOCE for functional assays. The pre-incubation of PMN with 2-APB prior to sporozoite exposure resulted in a significant diminishment of NETosis and therefore confirmed the assumption that parasite-triggered NET formation is a Ca^2+^-(SOCE)-dependent process.

Besides being SOCE-dependent, ROS production is up-regulated in a CD11b (CR3)-dependent manner in bovine PMN [31]. In turn, up-regulation of CD11b proves as SOCE-dependent since it is inhibited by 2-APB [32] leading us to the assumption that the neutrophil integrin receptor CD11b may also be involved in sporozoite-mediated NETosis. Indeed, we found significantly enhanced levels of surface CD11b expression on parasite-exposed bovine PMN. The fact that antibody-mediated blockage of CD11b additionally resulted in NET diminishment suggests CD11b to be involved in sporozoite-triggered NETosis and to function as a PMN-derived receptor in this effector mechanism. These findings were in accordance with Byrd et al. [21] who reported on CD11b as a potential ligand in NETosis since antibody-mediated blockage of this integrin inhibits fibronectin-dependent NET formation to *C. albicans* hyphae.

Moreover, SOCE is tightly regulated via ERK1/2 MAPK phophorylation, i.e., by a signaling pathway that was recently described to be involved in NETosis [35]. In addition, ROS-dependent activation of ERK and p38 MAPK was demonstrated to mediate PMA-induced NET release from human neutrophils [35]. In accordance to the latter reports and to data on other pathogens [12,21] we here show a sporozoite-triggered up-regulation of ERK1/2 and p38 MAPK phosphorylation in parasite-exposed bovine PMN. The key role of the ERK1/2- and p38 MAPK-dependent signaling pathway in NETosis was confirmed in functional inhibition experiments showing that parasite-triggered NETosis was significantly diminished in the presence of ERK-and p38 inhibitors.

We furthermore focused on the relevance of NE and MPO and confirmed the key role of these two molecules in *E. bovis*-triggered NETosis. As with other bovine pathogens [18], exposure to sporozoites led to an increase of both, NE and MPO enzymatic activities in bovine PMN. Functional inhibition assays confirmed the relevance of these enzymes since parasite-mediated NETosis was significantly blocked when NE and MPO inhibitors were applied. Finally, the co-localization of DNA-rich filaments with NE- or MPO-derived signals in sporozoite-induced NET structures emphasized the classical characteristics of NET. These results were in line with recent findings on other coccidian parasites, such as *E. arloingi* [16] or *B. besnoiti* [17].

Whilst NE and MPO originate from azurophilic PMN granules, other molecules such as metalloproteinases (MMP), are contained in tertiary (gelatinase) granules. We here analyzed whether MMP-9 is released from PMN upon sporozoite exposure. Zymographic analyses of PMN supernatants confirmed a parasite-mediated MMP-9-release into PMN supernatants. So far, the functional role of MMP-9 in NETosis is unclear. However, it is worth noting that the release of MMP-9 is regulated by the ERK 1/2 and p38 MAPK signaling pathway [36]. Interestingly, Carmona Rivera et al. [37] showed an impaired endothelial function induced by NET-externalized MMP. Given that *E. bovis* sporozoites infect and mature within endothelial cells, a yet unclear interrelation between NETosis and parasite development within endothelium may exist.

Overall, since the first description of NET as an innate effector mechanism [6], most studies have focused on NETosis driven by bacterial, viral and fungal pathogens and, less frequently, on parasitic pathogens [13]. Nonetheless, there is increasing evidence on the relevance of NET as a defense mechanism against protozoan infections in vitro and in vivo [12,13]. Thus, NET-related data are available on some protozoan pathogens, such as *E. bovis, Toxoplasma gondii, Plasmodium falciparum, E. arloingi,* and *B. besnoiti* [5,12,15-17] and *Leishmania spp.* [14]. The current data suggest NETosis as a generally valid effector mechanism against coccidian parasites. Thus, NET release occurred irrespective of the parasite stage (*E. bovis* sporozoites, merozoites I, oocysts), and PMN origin (bovine, caprine, equine, canine). In agreement, data on different *Leishmania* stages [14] also indicated NETosis as a stage-independent defense mechanism. In addition, *T. gondii*-triggered NETosis has been reported for both human and murine PMN [12] indicating host-independent reactions. Overall, it is noteworthy that the degree of entrapment clearly differed amongst different parasite species and stages. Thus, we here report on 43% of *E. bovis* sporozoites to be immobilized within bovine NET structures, whilst in the caprine system even 72% of *E. arloingi* sporozoites were trapped [16] but only 34% of *B. besnoiti* tachyzoites were found ensnared in NET being expelled from bovine PMN [17]. Since all these parasites exhibit an obligatory intracellular replication, extracellular immobilization via NET will have a tremendous implication on the outcome of the disease as previously postulated [16,17].

Furthermore, NETosis was not parasite species-specific since sporozoites of different coccidian parasites (*E. bovis*, *E. arloingi*, *I. suis*, *T. gondii*) equally triggered NET formation. To our best knowledge this is the first report on *T. gondii* and *I. suis* sporozoite-induced NET extrusion whilst tachyzoite stages of *T. gondii* [12] and sporozoites of *E. arloingi* [16] have already been reported before. Especially in the case of *T. gondii*, which exhibits an enormous proliferation at the tachyzoite stage, the intervention of the immune system at a very early time point, i.e. when sporozoites invade the host system and before non-sexual parasite replication occurs, will have a higher impact on the outcome of disease than tachyzoite-triggered reactions. Taking into account that PMN have been demonstrated to actively transmigrate into the intestinal lumen and were found alive in the gut mucus [38], it seems feasible to assume that they may interact with luminal pathogen stages, such as ingested *Eimeria* oocysts, in vivo. Consistently, we here show that oocysts of *E. bovis* are trapped and sometimes almost encaged by NET structures in vitro, thereby most probably preventing proper excystation of sporozoites. This novel anti-excystation mechanism of NET has recently been hypothesized for *E. arloingi* oocysts, in which sporozoites were impeded to escape from NET-covered preformed excystation sites (micropyles) of sporulated oocysts [16]. Thus, in the in vivo situation*,* luminal occurring NETosis might intervene very early after oral infection with the parasite and abrogate infection even before infectious stages (sporozoites) are able to evade immune reactions via intracellular positioning.

In summary, NET formation seems to be an ancient and highly conserved host effector mechanism of PMN acting against several pathogens as an early host immune reaction. In the present work, we added new data on the molecular mechanisms involved in parasite-triggered NETosis and call for more investigations on receptor-ligand-interactions.
